# Age-Related Dynamics of Circulating Innate Lymphoid Cells in an African Population

**DOI:** 10.3389/fimmu.2020.594107

**Published:** 2020-12-02

**Authors:** Alansana Darboe, Carolyn M. Nielsen, Asia-Sophia Wolf, Jacob Wildfire, Ebrima Danso, Bakary Sonko, Christian Bottomley, Sophie E. Moore, Eleanor M. Riley, Martin R. Goodier

**Affiliations:** ^1^ Department of Infection Biology, London School of Hygiene and Tropical Medicine, London, United Kingdom; ^2^ Vaccines & Immunity Theme, Infant Immunology, MRC Unit The Gambia at London School of Hygiene and Tropical Medicine, Fajara, Gambia; ^3^ Nuffield Department of Medicine, University of Oxford, Oxford, United Kingdom; ^4^ Division of Infection and Immunity, University College London, London, United Kingdom; ^5^ Nutrition Theme, MRC International Group, MRC Unit The Gambia at London School of Hygiene and Tropical Medicine, Keneba, Gambia; ^6^ Department of Infectious Disease Epidemiology, London School of Hygiene and Tropical Medicine, London, United Kingdom; ^7^ Women & Children’s Health, Kings College London, London, United Kingdom; ^8^ Institute of Immunology and Infection Research, School of Biological Sciences, University of Edinburgh, Edinburgh, United Kingdom

**Keywords:** innate lymphoid cells, age, CD117, IL-13, memory CD4+ T cells, Gambia

## Abstract

Innate lymphoid cell (ILC) lineages mirror those of CD4+ T helper cell subsets, producing type 1, 2 and 3 cytokines respectively. Studies in adult human populations have shown contributions of non-cytotoxic ILC to immune regulation or pathogenesis in a wide range of diseases and have prompted investigations of potential functional redundancy between ILC and T helper cell compartments in neonates and children. To investigate the potential for ILC to contribute to immune responses across the human lifespan, we examined the numbers and frequencies of peripheral blood ILC subsets in a cohort of Gambians aged between 5 and 73 years of age. ILC2 were the most abundant peripheral blood ILC subset in this Gambian cohort, while ILC1 were the rarest at all ages. Moreover, the frequency of ILC1s (as a proportion of all lymphocytes) was remarkably stable over the life course whereas ILC3 cell frequencies and absolute numbers declined steadily across the life course and ILC2 frequencies and absolute numbers declined from childhood until the age of approx. 30 years of age. Age-related reductions in ILC2 cell numbers appeared to be partially offset by increasing numbers of total and GATA3+ central memory (CD45RA-CCR7+) CD4+ T cells, although there was also a gradual decline in numbers of total and GATA3+ effector memory (CD45RA-CCR7-) CD4+ T cells. Despite reduced overall abundance of ILC2 cells, we observed a coincident increase in the proportion of CD117+ ILC2, indicating potential for age-related adaptation of these cells in childhood and early adulthood. While both CD117+ and CD117- ILC2 cells produced IL-13, these responses occurred predominantly within CD117- cells. Furthermore, comparison of ILC frequencies between aged-matched Gambian and UK young adults (25–29 years) revealed an overall higher proportion of ILC1 and ILC2, but not ILC3 in Gambians. Thus, these data indicate ongoing age-related changes in ILC2 cells throughout life, which retain the capacity to differentiate into potent type 2 cytokine producing cells, consistent with an ongoing role in immune modulation.

## Introduction

Innate lymphoid cell (ILC) lineages are defined by the expression of key transcription factors and production of cytokines previously associated with T cell lineages. The cytotoxic Group 1 ILC population includes conventional natural killer cells (cNK) and ILC1, while the functions of non-cytotoxic ILC mirror those of CD4+ T helper (Th) cell lineages. In contrast with T helper cells, however, ILC rely on accessory cytokines and/or non-polymorphic germ-line encoded receptors for their activation and regulation. Group 1 ILC (ILC1) respond to interleukin-12 (IL-12) and IL-18 to produce interferon-γ (IFN-γ). Group 2 ILC (ILC2) respond to localized stromal cell (IL-33 and thymic stromal lymphopoietin) or mucosal epithelial cell-derived factors (IL-25) to produce IL-5 and/or IL-13. Group 3 ILC (ILC3) are defined by RORγt and differential expression of NKp44 and either IL-22 or IL-17 with NKp44+ ILC3 being the main producers of IL-22 in tissues (1, 2).

ILC are present at high frequencies and in high absolute numbers in cord blood and in the peripheral blood of children (aged 6–18 years) and, more recently, frequencies of ILC2 and ILC3 cells have been seen to decline with increasing age in adults (3, 4). Functional redundancy between CD4+ T cell subsets and their ILC counterparts has been inferred, as ILC-related deficiencies do not lead to apparent disease (3). Redundancy between ILC and CD4+ T cells has also been demonstrated in murine infection models, e.g. by ILC2 activity influencing early responses to worm infection but disease ultimately being controlled by CD4+ Th2 cells (5).

Recent evidence has indicated that, like T cells, ILC can recirculate *via* the lymph and blood and home to specific tissues, where the local tissue microenvironment plays a role in their functional differentiation (6). Gene expression and RNA velocity analysis have demonstrated the relatedness of human tonsillar ILC1 and ILC3 with the potential for conversion of ILC3 into IFN-γ producing ILC1, in the presence of specific transcription factors and tissue microenvironments (7). Similarly, *in vitro*, human ILC3 can trans-differentiate into IFN-γ producing cells in the presence of IL-12 (+/- IL-15) and human ILC1-like cells can be derived from ILC3 after stimulation with IL-2 and IL-23; IL-1β reverses this process (8). Human blood ILC2 also demonstrate considerable plasticity and switch from IL-13/IL-15 to IFN-γ production after treatment with IL-12 + IL-1β (9–11).

Endogenous, tissue-derived signals are implicated in controlling the pattern of activating receptor expression on ILCs and, thus, controlling their function in different tissues and locations (12). Functional trans-differentiation of ILC2 has been associated with tissue-specific pathology. For example, in the upper airways, IL-4 mediates maintenance and proliferation of airway ILC2, whereas IL-12 mediates their differentiation toward ILC1. Thus, differences in local cytokine environments may underlie the observations that chronic obstructive pulmonary disease (COPD) is associated with reduced representation of ILC2 (and NKp44+ ILC3) and enrichment of ILC1, whereas, nasal polyps are associated with enhanced frequencies of ILC2 (9, 11). Similarly, the local cytokine environment may explain why intrahepatic ILC2 IL-13+ cells correlate with end stage liver disease (13).

More recently, two subpopulations of ILC2 (CD117- and CD117+) have been described. The behavior of the two subsets depends on the local tissue milieu or, *in vitro*, on polarizing conditions during activation and has implications for disease outcomes (14–16). Sampling of the upper airway mucosa in chronic rhinosinusitis has demonstrated that CD117-IL1R- ILC2 are present exclusively in individuals lacking nasal polyps, whereas CD117+ILC2 cells are associated with the presence of nasal polyps, Th2 cytokines and eosinophilia (17).

To investigate frequencies and functional potential of ILC subsets over the human life course, we examined changes in CD4+ T cell and ILC subsets in a cohort of West African (Gambian) children and adults. ILC2 (Lineage- CD161+ CRTh2+) cells were the most abundant ILC subset within this study population at all ages but progressively declined in frequency and number with age. However, the proportion of CD117+ ILC2 increased with age, consistent with maintenance of ILC2 plasticity throughout life.

## Materials and Methods

### Study Subjects

This study was approved by the ethical review committees of The Gambia Government/Medical Research Council Unit, The Gambia and London School of Hygiene and Tropical Medicine (SCC1449v2 and 6034). Subjects were recruited from the villages of Keneba, Manduar, and Kantong Kunda in West Kiang District, Lower River Region, The Gambia. After giving fully informed consent, including parental/guardian consent for minors, venous blood samples were collected from 652 individuals (425 females, 227 males) aged 5–73 years. Individuals with signs or symptoms of current infection or chronic disease or known to be pregnant or infected with HIV or Malaria, were excluded. For comparative phenotyping, 45 UK individuals (26 females; 19 males) between 25–29 years of age were recruited using the same study protocol as above.

### Peripheral Blood Mononuclear Cell Preparation and Culture

Peripheral blood mononuclear cell (PBMC) were isolated by density gradient centrifugation (Histopaque, Sigma, UK) and analyzed directly *ex vivo* or after *in vitro* stimulation. ILC activation was performed after overnight resting of PBMC followed by 6 h of stimulation with PMA + ionomycin (1:500 final concentration, cell stimulation cocktail, Thermo-Fisher, UK). Cytokine stimulation was performed on PBMC maintained overnight in IL-7 (20 ng/ml) and stimulated for a further 6 h with IL-33 and thymic stromal lymphopoietin (TSLP) (both at 50 ng/ml, Peprotec, UK). Intracellular cytokine staining was performed after adding Brefeldin A and Monensin (GolgiPlug and GolgiStop, respectively) 4 h prior to harvesting.

### Flow Cytometry

The following monoclonal antibodies were used to identify ILC subsets within PBMC: Lineage (lin) markers, anti-CD3-V500 (BD Biosciences, UK), anti-CD4-AlexaFluor 700 (Biolegend, UK) and FITC conjugated CD14, CD16, CD19, CD21, CD94, anti-CD11c, anti-CD123 and anti-BDCA 2 (all from eBioscience, Thermo Fisher, UK). ILC subsets were positively identified by staining with anti-CD127, anti-CD161, anti-CD117 (c-kit) and anti-CD294 (Chemoattractant receptor-homologous molecule expressed on Th2 cells- CRTh2) and sequential gating. The following additional panel of fluorochrome-conjugated monoclonal antibodies were used for surface staining in CD4+ T cell subset frequency analysis: CD4 FITC (eBioscience, UK), CCR7 PE-Cy7, CD45RA AF700 (both from Biolegend, UK) and after fixation and permeabilization with FOX-P3 staining kit (eBioscience, UK), subsequently incubated with GATA-3 AF647 (Biolegend, UK) for intracellular staining. Data from 39 individuals were excluded from the T cell phenotypic analysis due to a CD4 mutation which prevented recognition by the OKT4 antibody clone used (18). For intracellular cytokine staining, cells were fixed and permeabilised using a commercially available kit (BD Biosciences, UK) and after surface staining, incubated with anti-IL-5PE and anti-IL-13 PE-Cy7 (Both from Biolegend, UK).

Two million PBMC were stained directly *ex vivo* or for each *in vitro* culture condition stimulation. Cells were acquired on a LSRII^®^ flow cytometer using FacsDiva^®^ software (Becton Dickinson, UK. Data analysis was performed using FlowJo^®^ (TreeStar). Only samples with at least 300 gated ILCs were accepted for analysis.

### Serological Assays

Total IgE was measured using a commercial ELISA kit (Thermo Fisher, Invitrogen, UK) according to manufacturer’s instructions and using a seropositivity cut-off of 7.8 ng/ml. Ascaris antigen was kindly provided by MariaYazdanbaksh (Leiden, Netherlands) and used to estimate reactivity with serum IgG using an in-house protein microarray assay (19).

### Statistical Analysis

Statistical analysis was performed using Statview and Stata version 13.1. Non-linear effects of age were modeled using natural cubic splines in linear regression models; p-values (F-test) and R-squared values were obtained from these models. Differences between groups of individuals were compared using Wilcoxon signed rank tests.

## Results

### Significant Decline in ILC2 and ILC3 Numbers Throughout Life

**Figure 1:**
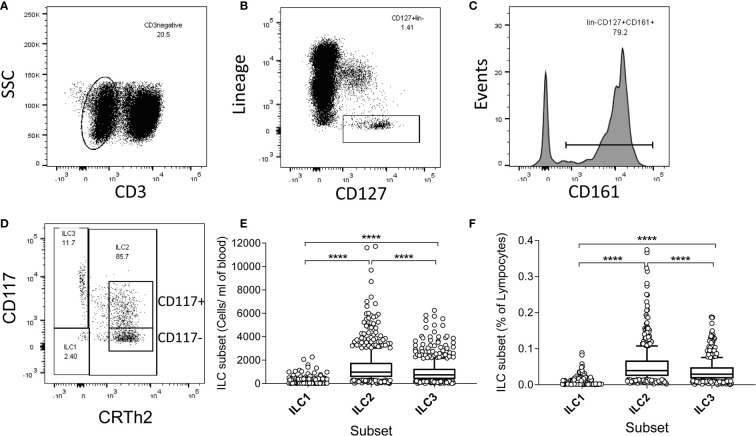
Gating strategy, and high absolute numbers and frequencies of innate lymphoid cell 2 (ILC2) in a Gambian population. **(A–D)** Flow cytometric gating strategy for human blood ILC subsets in 637 Gambian individuals. After initial gating on singlets and viable cells, peripheral blood mononuclear cell (PBMC) were sequentially gated on **(A)** non-T cells (CD3-), **(B)** lineage negative, CD127+ cells, **(C)** CD161+ cells and finally **(D)** ILC1, ILC2, and ILC3 subsets, according to the expression of CD117 and CRTh2. **(E)** Absolute numbers and **(F)** frequencies of ILC1-3 subsets. Box and whisker plots represent medians, 10–90^th^ percentiles and outliers. Differences in frequencies and absolute numbers between subsets were tested using one-way ANOVA with multiple comparisons. ****p<0.0001.

To examine whether high ILC frequencies are predominantly a feature of early life or are maintained and/or renewed over the life course, we measured ILC frequencies and absolute numbers directly *ex vivo* within isolated PBMC (**Figure 1**). ILCs were identified using sequential gating guided by a previously published protocol (20, 21), although with some differences: in our study, anti-CD4 was included in the lineage (lin) cocktail, potentially excluding CD4+ ILC1 cells in the analysis and TCRαβ and TCRγδ T cells were not excluded by our lineage cocktail panel. The gating strategy firstly excluded CD3+ T cells and lineage marker expressing cells and subsequently gating on CD161 and CD127 to identify ILC as lin- CD127+ CD161+ cells (20, 22) (**Figures 1A–C**). Within this population, ILC2 were identified as CD294+ (CRTh2) (CD117+ or -) cells, ILC1 as CRTh2- CD117- and ILC3 as CRTh2- CD117+ (**Figure 1D**). Overall, ILC2 were the most abundant subset both in terms of absolute number/ml of blood (median = 973/ml, 95% Confidence Interval (CI) = 905-1066/ml) and frequency within gated lymphocytes (median = 0.039%, 95% CI = 0.037-0.042%) (**Figures 1E, F**). ILC1 cells were the least abundant (median cells/ml blood = 108/ml, 95% CI = 107–115/ml; median % of lymphocytes = 0.0043%, 95% CI = 0.0041–0.0046%) with ILC3 cells being found at intermediate numbers (median = 741/ml, 95% CI = 675–789/ml) and frequencies (median = 0.029%; 95% CI = 0.027–0.031%) (**Figures 1E, F**).

**Figure 2:**
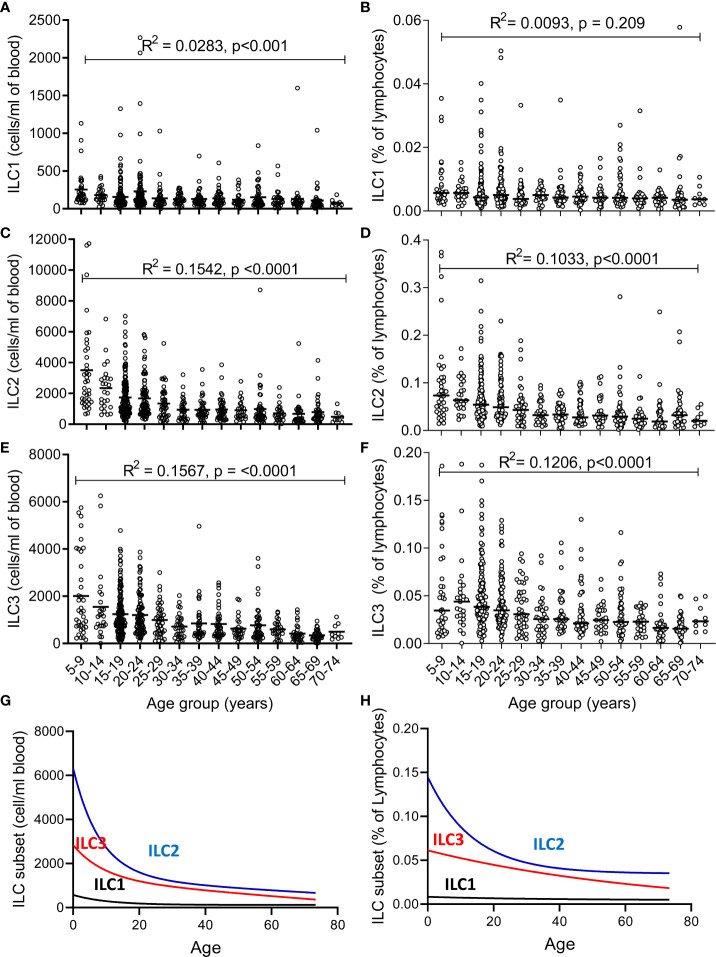
Changes in number and frequency of innate lymphoid cell (ILC) subsets over the lifespan. **(A–C)** Absolute numbers and **(D–F)** frequencies of **(A, D)** ILC1, **(B, E)** ILC2, and **(C, F)** ILC3 were estimated in 637 individuals aged between 5 and 75 years. Non-linear model of median value of ILC subset cell/ml blood **(G)** and % of lymphocytes **(H)** with increasing age. Data are shown as 5-year strata across the age span. Dots represent 637 individual values and bars represent medians. Analysis was performed using trend ANOVA. Significance (p-value) and R^2^ values are shown on each graph.

**Figure 3:**
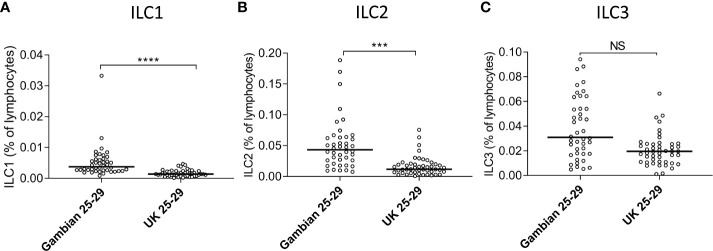
Age-matched Gambians adults have higher frequencies of innate lymphoid cell 1 (ILC1) and ILC2 than UK adults. Frequencies of **(A)** ILC1, **(B)** ILC2, and **(C)** ILC3 cells are shown within total lymphocytes. Comparison between Gambian and UK individuals (open circles) were made using Mann-Whitney U test. ***p<001, ****p<0.0001, Dots represent 45 Gambian and 45 UK individual values and bars represent medians. NS, not significant.

The absolute numbers of ILC1, ILC2, and ILC3 were highest in children and declined steadily throughout life (**Figures 2A–C**; note scale of y-axis varies between plots). However, although numbers of ILC1 cells declined with age they remained as a relatively constant proportion of the peripheral blood lymphocyte population, suggesting that the decline in ILC1 is predominantly driven by shrinkage of the lymphocyte pool with age (**Figure 2D**). In contrast, in addition to a decline in absolute numbers, ILC2 and ILC3 also declined as a proportion of total lymphocytes (**Figures 2E, F**). Application of a non-linear trend analysis to the frequencies and absolute numbers of ILC1, ILC2 and ILC3 subsets with age revealed distinct behaviors of these three subsets over the life course (**Figures 2G, H**). Consistent with dilution of the peripheral blood lymphocyte pool in early age, there was a significant decline in absolute numbers of all three ILC subsets between the age of 5 and 30 years (ILC1, p= 0.027; ILC2; p= <0.0001; ILC3 p=<0.001) (**Figure 2G**). After the age of 30 years, absolute numbers of ILC1 and ILC2 stabilized whereas numbers of ILC3 continued to decline significantly throughout life (p<0.0001) (**Figure 2G**). Similarly, frequencies of ILC2 declined up to the age of 30 years (p<0.0001) (**Figure 2H**) but stabilized thereafter whereas the age-related decline in the frequency of ILC3 cells continued throughout the lifespan, with a more significant negative trend occurring in individuals over 30 years (p<0.0001) than those under 30 years (p=0.015) (**Figure 2H**); no significant age trend was observed for frequencies of ILC1s cells (**Figure 2H**). In addition, comparison of ILC frequencies in young Gambian adults (25-29 years of age) with those of an aged-matched UK cohort revealed an overall higher proportion of blood ILC1 and ILC2, and a trend toward higher ILC3 frequencies in Gambian compared to UK donors (**Figure 3**).

### Age-Related Decline in ILC2 Parallels CD4+ T Effector Memory But Not CD4+ T Central Memory

**Figure 4:**
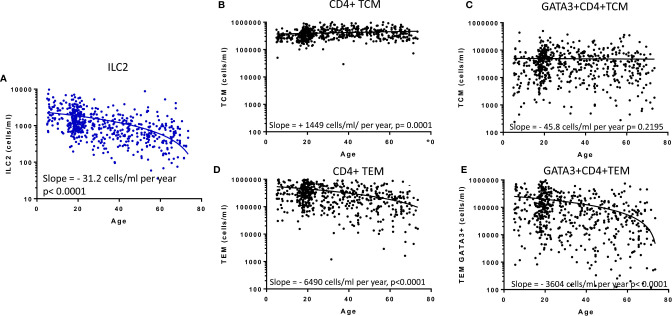
Relationship between memory CD4+ T cell subsets and innate lymphoid cell 2 (ILC2). The distributions of absolute numbers of **(A)** ILC2 (cells/ml blood), **(B)** T central memory (TCM), **(C)** GATA3+ TCM **(D)**, T effector memory (TEM) and **(E)** GATA3+ TEM (cells/ml blood) with age are plotted with non-linear regression lines shown. Dots represent 600 individual values across the age range.

Previous studies have implied redundancy between ILC and equivalent T cell subsets. We therefore compared the absolute numbers of ILC2 and memory CD4+ Th cell subsets to determine whether the age-related loss of ILC2 may be offset by acquisition of Th subsets (**Figure 4**). Accordingly, in contrast to the significant age-related decline in absolute numbers of ILC2 (**Figure 4A**), there is a significant increase in absolute numbers of total central memory T cells (TCM: CD45RA- CCR7+ CD4+) and maintenance of absolute numbers of Type 2 TCM (Th2 TCM: GATA3+ CD45RA-CCR7+ CD4+) (**Figures 4B, C**) across the life course. However, somewhat surprisingly, absolute numbers of effector memory T cells (TEM: CD45RA-CCR7- CD4+) and GATA3+ Th2 TEM declined significantly over the lifespan at rates similar to the decline of ILC2 cells (**Figures 4D, E**). These data suggest that turnover of ILC2 and effector memory Th2 cells may be co-regulated over the life course.

### Increased Frequencies of CD117 (c-kit) Expressing ILC2 Throughout Life

**Figure 5:**
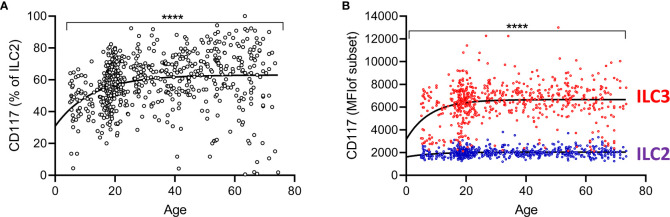
Increased representation of CD117+ innate lymphoid cell 2 (ILC2) with age. **(A)** Frequencies of CD117+ cells within total ILC2 are shown across the age range. Dots represent 637 individual values and the trend lines represent median values with increasing age. **(B)** Mean fluorescence intensities for CD117 on gated ILC2 and ILC3 subsets. Change in the frequency of CD117+ILC2 and MFI of CD117 on ILC2 and ILC3 cells with age was tested by trend ANOVA, ****p<0.0001.

Recent studies have indicated ILC2 can be divided into two functionally distinct subsets according to the expression of the stem cell factor receptor CD117 (c-kit) (15, 17). The majority of CD117+ blood ILC tend to be ILC3 cells (CRTh2-) and in our cohort the majority of CD117^hi^ cells were indeed ILC3 (**Figure 1D**). However, we also observed that a substantial proportion of ILC2 were also CD117+ (**Figure 1D**) and thus, in view of the abundance of ILC2 cells within this African cohort, we analyzed the frequencies of CD117+ ILC2 within this compartment throughout life. Overall, 60.9% of ILC2 were CD117+, inter quartile range (IQR), 49.8–69.9%. Surprisingly, a non-linear increase in the proportion of ILC2s that express CD117+ was observed with age, with median 48%, (IQR, 37.9-55.0%) of ILC2 being CD117+ in 5–9 year old children and maximal frequencies of these cells being found by 30 years of age (median 64%, IQR 55.2–69.1%) (**Figure 5A**). Similar to changes in their overall frequency, a significant increase in relative expression (Mean Fluorescence Intensity-MFI) of CD117 on ILC2 cells was observed with increasing age up to 30 years of age (p<0.0001) with no significant trend thereafter, indicating that CD117 acquisition on ILC2 cells could potentially contribute to enrichment of the CD117+ subset (**Figure 5B**). Similarly, a significant age-associated trend for increased CD117 MFI was observed for ILC3 cells among individuals under the age of 30 (p<0001) and continued to increase to some extent after 30 years of age (p=0.033) (**Figure 5B**). We also observed significantly higher frequencies of CD117+ ILC2 in females compared to males (Females: Mean 60.5%, standard deviation (SD) ±14.3%; Males: Mean 57.1%, SD ±16.0%, p=0.006) although there was no difference between female and male children under 10 years of age (data not shown), suggesting a potential impact of the onset of puberty on ILC phenotype. In summary, therefore, these data indicate that CD117+ ILC2 cells are found in the peripheral blood at high frequency across the life span and with the highest levels in adults.

### Functional Capacity of ILC2 Subsets in Children and Adults

**Figure 6:**
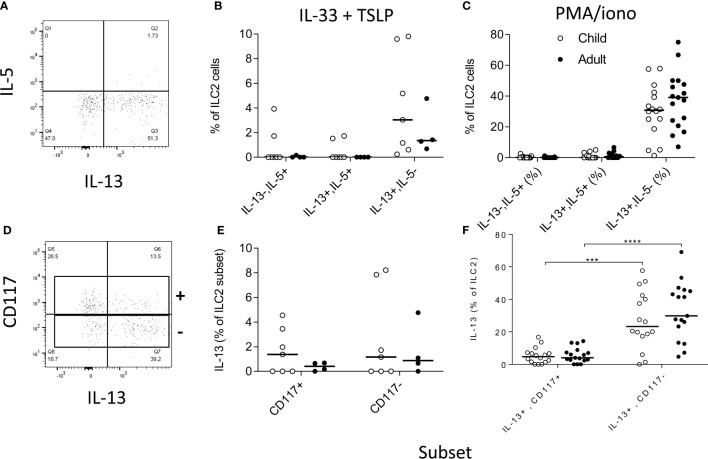
Functional capacity of innate lymphoid cell 2 (ILC2) subsets. Gating strategies for **(A)** cytokine responses within total ILC2 cells and **(B)** for IL-13 production by CD117 defined ILC2 subsets and frequencies of IL-5 and/or IL-13 producing cells after stimulation with **(B, D)** IL-33 + thymic stromal lymphopoietin (TSLP) or **(C, F)** PMA + ionomycin. Open circles represent responses in children (**B**, **E** n=7; **C**, **F** n=15) and closed circles represent responses in adults (**B**, **E** n= 4; **C**, **F** n=17). Comparison of cytokine production between subsets was performed using Mann-Whitney U test, ***p<0.001, ****p<0.0001.

We next investigated the functional potential of ILC2 in a randomly selected subgroup of children (n=15; mean age ± SD = 9.1 ±2.8 years) and adults (n=17; 22.8 ±3 years). To preserve the functional potential of distinct ILC subsets, we rested PBMC overnight and then stimulated them for 6 h with cytokines (IL-33+TSLP) or with PMA + ionomycin (**Figures 6B, C, E, F**, respectively). We detected IL-13 production by significant numbers of ILC2 cells, with only occasional co-expression of IL-5 (**Figures 6A–F**). There was no significant difference between children and adults in the capacity of ILC2 to produce IL-13 (**Figures 6B, C**) but IL-13 production was much more frequent among CD117- ILC2 than CD117+ ILC2 (**Figure 6F**) after PMA + ionomycin stimulation (although not after IL-33+TSLP stimulation) (**Figure 6E**).

### Relationship Between ILC2 Dynamics, Plasma IgE and Anti-Ascaris IgG

**Figure 7:**
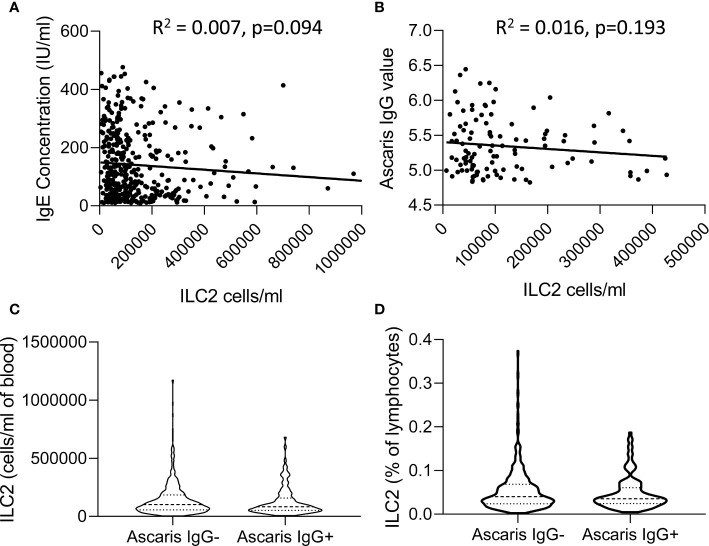
Relationship between total IgE or Ascaris-specific IgG concentrations and innate lymphoid cell 2 (ILC2) numbers. Linear regression of **(A)** Total serum IgE concentration and **(B)** anti-Ascaris IgG levels are plotted against the absolute number of ILC2 cells/ml blood. Violin plots showing comparison of **(C)** absolute number and **(D)** frequencies of ILC2 cells in anti-Ascaris IgG- and IgG+ individuals, each dot represents an individual, (n=637).

In view of previous reports of the impact of active helminth infections on ILC2 frequencies in African populations (14, 23), we investigated the relationship between ILC2 frequencies and two markers of exposure to helminth infection, namely plasma IgE and anti-Ascaris IgG concentrations (**Figure 7**). Although there was a trend toward a modest negative correlation between ILC2 numbers and both total IgE and anti-*Ascaris* spp. IgG concentrations, neither trend was statistically significant (**Figures 7A, B**). Similarly, we observed no significant difference in either absolute numbers or frequencies of ILC2s between *Ascaris-*exposed (IgG seropositive) and *Ascaris-*unexposed (IgG seronegative) individuals (**Figures 7C, D**) suggesting that exposure, at least to this parasitic helminth, had limited long term impact on the ILC2 compartment.

## Discussion

Understanding the dynamics of lymphocyte populations is essential for better understanding the balance between innate and adaptive immunity across the lifespan and for predicting the consequences of aging on immune function. Here we have described, the age-related dynamics of innate lymphocyte populations, observing age-related reductions in both the frequencies and absolute numbers of all ILC subsets across the lifespan. ILCs represent only a small proportion of all lymphocytes at any age, making up approx. 0.3% of lymphocytes in early life and approx. 0.05% in later life in this population. Although our study is limited by a lack of data from children under the age of 5 years, we can clearly see that while the total lymphocyte pool shrinks (at least in terms of cells/ml of blood) with age, ILCs are proportionally more affected by this change than adaptive lymphocyte populations. While this may reflect a gradual shift in emphasis of the immune system toward adaptive immune responses with increasing antigen experience, it is notable that the total number of circulating memory T cells changes very little with age, at least after the age of 5 years. The age-related emergence of central memory CD4+ T cells seems to be offset by a steady age-related decline in numbers of circulating effector memory CD4+ T cells. Given the likely high antigen load experienced by this rural African population it is possible that memory T cell populations mature quite rapidly in early life, that the available T cell niches are rapidly filled and that ongoing homeostatic constraints lead to retention of central memory cells at the expense of effector cells. It would be of considerable interest to compare rates of lymphocyte maturation between populations in different environments; the limited comparison shown here, between young Gambian and UK adults, suggests that there may indeed be marked differences between populations in the effect of age on lymphocyte distribution.

Although absolute numbers decline with age, the distribution of subsets within the ILC population seems to be quite stable after the age of 5 years, with ILC2 being the most abundant subset and ILC1s being the least abundant population at all ages. Despite being the least abundant ILC subset, the overall proportion of ILC1 cells among all lymphocytes does not decline significantly with age whereas the proportions of both ILC2 and ILC3 decline quite markedly. Although our gating strategy excluded a potential contribution of CD4+ ILC to blood ILC1 dynamics, these data are entirely consistent with a recent study reporting stability of ILC1-like cell frequencies between cord and peripheral blood and in peripheral blood lymphocytes from younger and older adults, contrasting with a marked reduction in the frequencies of ILC2 and ILC3 cells with increasing age (4, 21). Renewal of lymphocyte populations is dependent, in part, on expression of the stem cell growth factor receptor/tyrosine-protein kinase c-kit (CD117). ILC1 are defined, among other traits, by their lack of expression of CD117 and ILC3 by their high levels of CD117 expression (20). By contrast, CD117 expression varies markedly among ILC2 with a distinct population of CD117- cells and a more diverse population of CD117+ ILC2. The proportion of CD117+ ILC2 increased with age in this Gambian population and we also noted a significant upregulation of CD117 expression (i.e. increased MFI) on both ILC2 and ILC3 cells in younger individuals. ILC2 and ILC3 derive, independently, from CRTh2- NKp44- CD127+ c-kit (CD117)+ precursors and NKp46+ c-kit+ precursors expressing CD56+, respectively (24). Although the relatedness of CD117+ and CD117- cells within the CRTh2+ ILC2 population has not been directly established, the former express CXCR6 and have superior trans-differentiation potential under both ILC1 and ILC3 polarizing conditions (15). Importantly, GATA3 expression is lower in c-kit+ compared to c-kit- ILC2 indicating a potential role for this transcription factor in the lineage relationship and plasticity of these subsets. Indeed, *in vitro* culture of ILC2s with type 2 polarizing cytokines reduces the frequency of c-kit+ CXCR6+ relative to c-kit- ILC2 cells and stimulation with SCF enhances the frequency of IL-13 producing c-kit^lo^ ILC2s.

While we have examined only peripheral blood ILC, there is limited evidence that circulating blood ILC precursors may give rise to tissue ILC in response to local stimuli (6) and, despite limitations in quantitative comparison of cells in blood and tissues, there is evidence that ILC2, ILC3 and NK have a relatively similar subset distribution in blood, non-mucosal tissues (except for the lung) and the skin (25). In contrast, ILC3 are enriched relative to ILC2 and NK cells in mucosal tissues. However, in that study, ILC1 were reported as virtually in tissues, with the exception of an ILC1-intrepithelial -like subset in some mucosal tissues (25).

The precise signals leading to differentiation and expansion of ILC are debated. ILC2 are already present at high frequencies in cord blood and it is therefore likely that intrinsic factors are involved in their early differentiation and expansion (3) and, postnatally, diverse infection-independent factors and cellular interactions have been found to activate or co-stimulate ILC2 potentially leading to their maintenance or expansion [reviewed in (26)]. IL-33 activates human ILC in the presence of costimulatory factors or cytokines belonging to the common γ-chain family (IL-2, IL-4 IL-7, IL-9, TSLP). Prostaglandin D2 (PGD2) specifically induces migration of blood and skin CRTh2+ ILC2, indicating that ILC2 localization may be influenced by tissue PGD2 concentrations and/or their levels of expression of the PGD2 receptor, CRTh2 (27). Interestingly—given our observation of sex differences in ILC numbers in individuals over the age of 10 years, but not in those under 10 years of age—recent studies in mice have suggested that ILC2 numbers may be influenced by androgen concentrations, with a sex bias in ILC numbers occurring after reproductive age (28). The roles of prostaglandins and sex hormones in the differentiation, maintenance and function of ILC merit further investigation.

Despite evidence for lifelong renewal of ILC subsets, their numbers and proportions do decline with age. Interestingly, the trajectory of decline in numbers of circulating ILC2 is almost identical to that of GATA3+ CD4+ effector memory T cells (TEM), raising the possibility that the turnover or tissue migratory properties of these two subsets of lymphocytes may be co-regulated. Despite their falling numbers, TEM are reported to proliferate at a higher rate than central memory T cells (TCM), consistent with a much higher turnover, more frequent replenishment and shorter half-life of TEM compared to TCM (29). It is feasible that antigen specific encounters drive only occasional proliferation of TCM, whereas both TEM and ILC2 may have lower thresholds for activation and thus turn over more rapidly.

Finally, we compared the functional attributes of ILC2 in children and adults. In line with published data (15), we observed that CD117- (c-kit^lo^) ILC2 are very efficient producers of IL-13. IL-13 production by ILC2 did not seem to differ between children and adults but, due to limitations of cell numbers, we were only able to compare children aged 8-12 years with young adults (20-25 years) and it remains possible that functional differences exist at either end of the age spectrum. Nevertheless, our data suggest that CD117- ILC2 represent a small pool of cells with lifelong potential for production of IL-13; such data are also consistent with a previously proposed functional equivalence of GATA3+ CD4+ Th2 cells and ILC2 (3). Given this functional equivalence, it is tempting to ascribe the relative abundance of ILC2 in this population to exposure to helminth parasites. However, ILC2 numbers were not significantly correlated with total IgE concentrations, anti-*Ascaris* spp. IgG concentrations, or anti-*Ascaris* seropositivity. Although interpretation of this data is limited by the cross sectional nature of our study, it is in broad agreement with a study of Zimbabwean children where active helminth infections (as determined by *Schistosoma* spp. egg count) were associated with low frequencies of ILC2 and ILC2 numbers increased after treatment; the authors concluded that high frequencies of peripheral blood ILC2 can occur independently of worm infections (23). On the other hand, Lin−/CD45+/ckit+/CD127+ cells with capacity for IL-4, IL-5 and IL-13 production were found to be enriched in individuals with filarial infections, although these cells were not stained for CRTh2 and thus cannot be formally defined as either ILC2 or ILC3 (14). These diverse conclusions may simply reflect differences in study design or may indicate that different type 2 immune response-inducing infections may differ in their effects on ILC2 cells. In mice, resistance to *Nippostrongylus brasiliensis* (hook worm) infection is IL-33 dependent, ILC2/IL-13 mediated and IL-4 independent (5) whereas in *Heligmosomoides polygyrus* infections, nematode-derived factors can suppress IL-33, interfering with ILC2 activation (30). Redundancy between type 2 CD4+ T cell and ILC2 cell compartments and dominance of Th2 derived IL-4 mediated effects on humoral responses could also explain a lack of relationship between ILC2 frequencies and total IgE or anti-Ascaris IgG levels.

In conclusion, although ILC are more abundant in early life than in later years, our data are consistent with a scenario in which blood CD117+ ILC2 are preferentially maintained compared to their CD117- counterparts. The functional benefits of reducing the overall representation of this small population of IL-13-producing ILC2s are unclear but may well be related to adequate representation of type 2 T helper cell-mediated immune memory. Better understanding of the tissue tropism and distribution of these ILCs and their response kinetics relative to GATA3+ Th2 TCM and TEM populations may go some way toward addressing this question. These data highlight the need to further investigate the role of ILCs in adulthood and their potential contributions to immunity to infection, allergic reactions and inflammatory diseases.

## Data Availability Statement

The raw data supporting the conclusions of this article will be made available by the authors, without undue reservation.

## Ethics Statement

The studies involving human participants were reviewed and approved by The Gambia Government/Medical Research Council Unit, The Gambia and London School of Hygiene and Tropical Medicine (SCC1449v2 and 6034). Written informed consent to participate in this study was provided by the participants’ legal guardian/next of kin.

## Author Contributions

AD, CN, A-SW, JW, ED, and MG processed the samples and AD, CN, A-SW, and MG performed the experiments. BS managed the Gambian dataset and CB assisted with the statistical analyses. MG conceptualized the study protocol. SM, ER, and MG designed the project. AD, ER, and MG wrote the manuscript. All authors contributed to the article and approved the submitted version.

## Funding

This work was jointly supported by the U.K. Medical Research Council (MRC) and the U.K. Department for International Development (DFID) under the MRC/DFID Concordat agreement (G1000808).

## Conflict of Interest

The authors declare that the research was conducted in the absence of any commercial or financial relationships that could be construed as a potential conflict of interest.

## Abbreviations

ILC, innate lymphoid cells; TCM, CD4+ T central memory; TEM, CD4+ T effector memory.
